# A roadmap to good practice for training supervisors and leadership: a European perspective

**DOI:** 10.3389/frma.2025.1531467

**Published:** 2025-06-19

**Authors:** Seán Lacey, Tamarinde Haven, Rita Santos, Tom Farrelly, Máiréad Murray, Panagiotis Kavouras

**Affiliations:** ^1^Munster Technological University, Cork, Ireland; ^2^Department of Methodology and Statistics, School of Social and Behavioral Sciences, Tilburg University, Tilburg, Netherlands; ^3^Department of Ethics, Law and Humanities, Amsterdam UMC, Amsterdam, Netherlands; ^4^Health Services Executive, Cork, Ireland; ^5^HEAL-Link Office, Library & Information Center, Aristotle University of Thessaloniki, Thessaloniki, Greece

**Keywords:** training supervisors, leadership, research integrity, open science, research culture

## Abstract

**Purpose:**

Supervision and leadership are regarded to have a major role in promoting responsible research. Various approaches to training for supervisors and leaders have been proposed. However, little is known about what works best, what kind of hurdles are faced in implementation and engagement, and what methods of assessing the effectiveness of training programs are available. Through exploring these points, this research aims to propose a roadmap to good practice for training supervisors and leadership.

**Design:**

A virtual marketplace for exchanging current practices and approaches for training supervisors and leadership took place in March 2024. Twenty-two policy makers from thirteen European countries, supervisors and senior research leaders were selected to participate, using opportunistic and purposive sampling. Facilitated using the Gather platform, the marketplace commenced with a non-European keynote speaker on training supervisors and leadership. Three main questions were brought forward for discussion separately—*What works well for successful implementation*? *What are the challenges*? *How do we assess effectiveness*? After the keynote presentation, marketplace participants rotated in groups between three market stalls to share thoughts on good practices for training supervisors and leadership framed around the three questions. Moderators for each of the stalls recorded detailed field notes to inform the study findings.

**Findings:**

During the exchange, mandatory training, especially when tailored to specific disciplines and conducted in small groups using a problem-based learning approach, was deemed effective. Awareness of power imbalances between early career researchers, supervisors, and leaders were to the fore. Critical challenges included a need for senior supervisors and leaders to participate and support research training. Also a need for systemic processes, tailored to specific local settings to avoid *ad hoc* implementation of policies, procedures and training. In assessing effectiveness there was an emphasis to share more research data and to utilize incidents of breaches of research integrity. The latter to be leveraged for learning purposes and transparency around the investigative process.

**Originality:**

There are multiple facets to good practice for training supervisors and leadership, along with a multitude of practices, however there is little evidence of practices that work, challenges around implementation, and assessing effectiveness.

## Introduction

An important suggestion to promote responsible conduct of research is to equip supervisors and leadership with the necessary skills against the backdrop of international research policy changes within Higher Education Institutions (HEIs) and Research Performing Organizations (RPOs). There is a significant amount of literature on leadership in education (Senge et al., 2014; Murphy et al., 2006), along with literature on responsible research (Forsberg et al., 2018; Curry et al., 2020), but minimal literature on good practices for training supervisors and leadership. There are many challenges in research, where strong leadership is required, including counteracting pressures to publish, seeking innovation and impact, acquiring research funding, addressing instances of research misconduct and questionable research practices (QRPs; Evans et al., 2022; Horbach et al., 2022), however there is little evidence in the literature as to training and evidence around what works when it comes to informing research supervisors and leaders on good practices in their leadership. This speaks to elements of the research culture within HEIs and to the importance of training and upskilling, along with challenges of embedding good research practices.

Research integrity (RI) challenges and pressures are wide and varied. Wellcome Trust (2020) states that researchers that work in the areas of RI and Open Science are not always recognized by funding agencies, while, in many cases, the culture of “publish or perish” remains a staple to the potential detriment of value and substance (Neill, 2008; Rawat and Meena, 2014). The current methods of research assessment put pressure on researchers to target individual performance metrics, without paying much attention to the impact on supervising and leadership, society and the wider community (Curry et al., 2020). Curry et al.'s (2020) working paper outlines emerging forms of research assessment, including but not limited to, the San Francisco Declaration on Research Assessment (DORA; Declaration on Research Assessment, 2012), the Leiden manifesto (Hicks et al., 2015), the Hong Kong Principles (Moher et al., 2020) and Coalition for Advancing Research Assessment: CoARA (Science Europe, European University Association, and Stroobants, 2022). These forms of research assessment with a focus on qualitative assessment of research outputs as opposed to quantitative are a shift away from the “publish or perish” culture. However, there is little evidence on successfully implementing these new forms of assessment and on the positive impacts they may have on the promotion of responsible research. Hence, it is necessary to train supervisors and research leaders on responsible research.

Whereas training for PhD candidates in good practices in research has been implemented across Europe, with examples in some countries of mandatory training (e.g., Netherlands Code of Conduct for Research Integrity, 2018, p. 20), the implementation of training for PhD supervisors and research leaders is less uniform and often non-compulsory, and in some countries there is a lack of guidance and training at any level (European Commission, 2019). Casci and Adams (2020) wrote of the importance of “develop[ing] policies, guidance, communications, training and related initiatives that support the success of researchers at all stages of their career” (p. 1). However, they follow this by stating that developing policy is not enough, researchers should be trained and shown how to implement and make practical changes to their research practices. Taswell (2023) goes a step further by questioning the meaning and purpose of policies and procedures if the rules are not imposed and enforced for all members of the university.

The need for training is recognized widely (Forsberg et al., 2018; Pizzolato and Dierickx, 2023) and various proposals for the contents of training have been issued (Plemmons and Kalichman, 2017; Haven et al., 2022). In Europe, the League of European Research Universities (consisting of 24 universities) has mandated training for PhD supervisors in RI (Lerouge and Hol, 2020). In addition, the revised ALLEA European Code of Conduct for RI explicitly states that researchers, including the upper level of seniority, receive training in responsible research (All European Academies, 2023). Furthermore, the Ministry of Higher Education and Science (2014) states that a “fundamental part of sustaining and developing a culture of research integrity is the role of supervisors and senior researchers acting as mentors and role models. Thus, it is important that supervisors and senior researchers engage in research integrity teaching, training, and supervision” (p.16). Fortunately, some guidelines on what should be included in educating senior researchers have been developed (Tijdink et al., 2023), but there is a lack of consensus across jurisdictions.

Murphy et al. (2006) posits that leadership can be shared among multiple stakeholders with clear relationships between its leaders and the wider community. Work from Hanover Research (2014) on recommendations for embedding good practices within the research culture builds on this and found that establishing congenial relationships among researchers. This aligns with Senge et al.'s (2014) broad ranging work on collective leadership, highlighting the importance of collaborating with other leaders to ensure consistency. Within a HEI there may be many hierarchy levels—Departments within Schools; Schools within Faculties; Faculties within Campuses; Campuses within the HEI—that all wish to be distinct, but are required to comply with a core set of values, policies, and procedures analogous to a coupled system (Weick, 1976; Orton and Weick, 1990). Senge et al. (2014) advocate for being proactive, as opposed to reactive, when it comes to expanding focus by developing conditions that are sustainable and lead to change in the long run.

Singh (2001) and Mowles et al. (2018) share the view that when it comes to the higher education landscapes, there are continuous changes, with Mowles et al. (2018) reporting that the changes can be driven by external agencies, national and international policies, organizational structures and stakeholder engagement. In Wellcome Trust (2020) researchers were asked “What does good [research] culture look like?”, with one of eight most common responses being where “leadership is transparent and open” (p. 48). Interestingly from the same study, a suggested disconnect was found in supervisors' perceptions of their leadership and the reality. The authors posit that they do not know what drives this difference, but that supervisors and leaders may not “know what good looks like if they have not experienced it themselves or taken part in training, or if they do not regularly seek feedback from the people they manage” (p. 22).

While there is a significant volume of literature on leadership metrics in diverse fields (Sonmez Cakir and Adiguzel, 2020; Dael et al., 2022; Macfarlane et al., 2024), the current study's focus is on the effectiveness of training regarding RI for supervisors and research leaders. Considering the limited research on the effectiveness of training, this study aims to share experiences from European research policy makers, supervisors and senior research leaders as to what works best in training for supervisors and leaders, what kind of hurdles are faced in implementation and engagement, and what methods of assessing the effectiveness of training programmes are available. Alongside Evans et al. (2022), this study defines senior research leaders as being analogous to Professors and Heads of Department, which would equate to at least 5 years of experience working in research. To outline a roadmap to good practice for training supervisors and leadership, the study asked three related research questions: (1) What works well in terms of successful implementation and scaling up of good practice initiatives? (2) What are the challenges that researchers should be aware of, so that they can prepare to overcome? and (3) What would be meaningful ways to assess the effectiveness of implementing good practice?

## Materials and methods

### Ethical considerations

Research ethical approval was sought (SL) to carry out the study from the Munster Technological University Human Research Ethics Committee (MTU-HREC-MR-23-058-A).

### Literature search

A stepwise approach was adopted to distill literature available for this study (SL, TH). British Educational Index, Education Resources Information Center, Google Scholar and ScienceDirect were used to search for the keywords: training supervisors; research leadership; research integrity; research culture; and higher education. The European Commission, Nature and Science Europe were also searched for national and international RI policy and practitioner contexts. Literature searches were refined based on language being English and countries typically being in Europe and North America. Dates of publications were predominantly the past 20 years, although there were some older studies based on that literature being seminal work in the area.

### Methodological framework and design

With minimal evidence in the literature on the study's topic, a phenomenological paradigm with a primary qualitative data collection method was deemed appropriate (Connolly, 2016). This paradigm was chosen to enable the opportunity to build a rapport with participants, while being cognizant that the research data collected may not be representative of the population and may not be generalizable (Thomas, 2017). This paradigm was combined with the use of primary qualitative data to gain initial insights and function as a starting point when exploring the under-researched topic in an innovative group setting of a virtual marketplace. Demographic information on participants was solicited through an anonymous survey before the marketplace (see Appendix). These included participants sharing their role in research, whether they carry out research and their main place of work in Europe.

### Sample and participants

Using purposive sampling, professionals tasked with developing policy around training, designing and delivering training programs, and in charge of implementing and participating in training programs were identified. To ensure breath in the types of perspectives harvested, the maximum diversity in terms of European countries was aimed for (Patton, 2002). To obtain the sample, European research policy makers, supervisors and senior research leaders were identified based on the authors' professional network and membership of the Network for Education and Research Quality (NERQ),^1^ representing opportunistic and purposive sampling, but with a focus on geographical and stakeholder diversity. NERQ is a dedicated initiative (formed in 2022) that aims to enhance the quality and integrity of research through collaborative efforts with stakeholders in the research training community internationally. Potential participants were contacted via email over January and February 2024 (TH and SL). Based on the structure of the virtual marketplace, the plan was to have 20–24 participants (seven to eight participants per stall; Patton, 2002). In total 46 colleagues across 25 European countries were contacted with one reminder email sent in the case of no response. From the 46 invitations, 13 declined, 11 did not respond and 22 accepted the invitation with return of the informed consent form.

A minimal volume of demographic information was obtained from participants to provide context but maintain anonymity in findings due to the small scale of the study. Participants' main place of work was spread across 13 countries in Europe ranging from Austria to Greece to Slovenia and Spain (Table 1a). Sixteen (72.7%) participants were active in research at the time of the study. In addition to participants being European research policy makers, supervisors and research leaders, their role in their respective HEIs/RPOs varied from academics to research managers to ombudsperson and researcher (Table 1b).

**Table 1:** Demographic attributes of participants in terms of (a) Main place of work, and (b) Role in respective HEIs/RPOs.

**a**		
**Country**	**Frequency**	**Percent**
Austria	2	9.1
Belgium	2	9.1
Croatia	2	9.1
Denmark	2	9.1
France	2	9.1
Germany	1	4.5
Greece	2	9.1
Hungary	1	4.5
Ireland	4	18.2
Slovenia	1	4.5
Spain	1	4.5
Sweden	1	4.5
The Netherlands	1	4.5
Total	22	100.0
**b**		
**Role**	**Frequency**	**Percent**
CEO	1	4.5
Lecturer/Assistant professor	1	4.5
Ombudsperson related	3	13.6
Postdoctoral student	3	13.6
Professor	1	4.5
Research manager	6	27.3
Senior lecturer/Associate professor	2	9.1
Senior researcher/scientist	4	18.2
Trainer	1	4.5
Total	22	100.0

### Setting

The Gather platform^2^ was used to facilitate the virtual marketplace with a moderator for the event (TH). Dr. Daniel Barr—Principal Research Integrity Advisor, Research Strategy and Services, Research and Innovation, RMIT University, Melbourne, Australia—provided a keynote plenary presentation on training supervisors and leadership, with the impetus of proposing three main questions to be mapped to the function of stalls in the marketplace (analogous to breakout rooms in a virtual meeting). After the keynote speaker's presentation, and similar to a World Café (Wellcome Trust, 2024), market participants rotated between one of three market stalls to share thoughts on good practices for training supervisors and research leaders. Marketplace administrative and technical support was provided by LBOF and SL.

### Data collection

**Figure 1:**
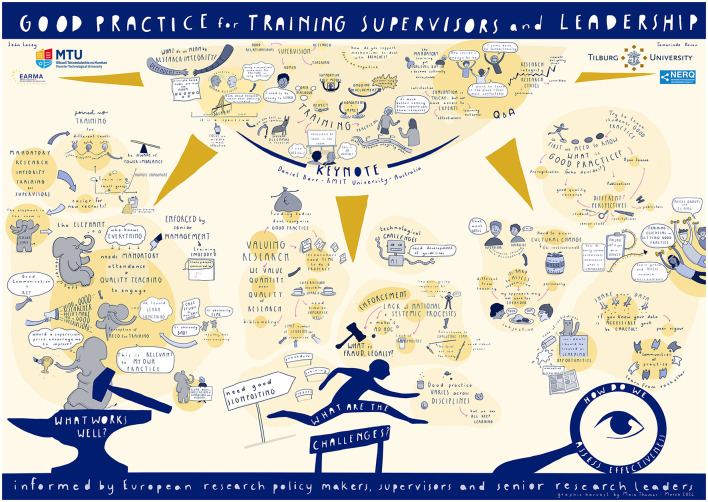
Graphic harvest summarizing the outputs from the study procedure of hammer and anvil, hurdle and magnifying glass (Haven and Lacey, 2024).

Each stall had a rapporteur (RS, TF, MM) who after allowing participants to introduce themselves, recorded the input of participants on the Gather platform's in-built whiteboard Eraser application. Participants had oversight of the notes from the whiteboard and were able to comment and propose edits during the marketplace for fairness and accuracy. When the second and third groups of participants entered a stall, the rapporteur informed participants of what was covered by the previous group(s), so that new information could be added in the discussion, as opposed to the same points being repeated. Furthermore, a graphic harvester (MT) migrated between stalls randomly gathering data to be included in a graphic harvest summarizing the main outputs from the marketplace (Figure 1; Haven and Lacey, 2024). Participants had oversight of the graphic harvest in draft form at the end of the marketplace with an opportunity to provide feedback, if they felt data collection may have been misrepresented.

### Procedure

The virtual marketplace had three stalls:

1. A “hammer and anvil” stall to be a platform for discourse on what works well in terms of successful implementation and scaling up of good practice initiatives.
2. A “hurdle” stall to act as a forum for discussion on challenges that researchers should be aware of, so that they can prepare to overcome.
3. A “magnifying glass” stall that was an area for researchers to share and discuss what would be meaningful ways to assess the effectiveness of implementing good practice.

When all participants had engaged with the three stalls, there was a return to a plenary where the three rapporteurs summarized the discussion points from their stall. With the small sample size, there are the potential biases that result from self-reporting of participant practices and experiences with responses to the three stall questions. However, as a small-scale study, this enquiry was intentionally designed as exploratory; not seeking to be definitive or representative, it instead aimed to uncover initial insights on this hereto under-researched area to inform future research.

### Data analysis and findings

Data was collated and analyzed (SL, TH) from whiteboard Eraser application, field notes and the graphic harvest to form the findings for this study. The data analysis was guided by the three research questions and was stopped when the full research team (SL, TH, RS, TF, MM, PK) agreed upon the finding's accuracy and comprehensiveness, reaching a consensus on the interpretation of the data contained in the rapporteur notes and graphic harvest.

### Research team and reflexivity

The research study was led by SL (PhD, male, extensive experience with research study support and data analysis) and TH (PhD, female, extensive experience with focus groups and interviews). RS (PhD, female, experience with focus group and moderating dialogues), TF (DEd, male, extensive research experience with a strong focus on qualitative methodologies), and MM (MSc, female, extensive experience with research study support and data management) moderating the dialogue in the stalls. With some of the research team facilitating aspects of the virtual marketplace, insider researcher positionality may be a perceived concern in research findings (Thomas, 2017). To mitigate against potential researcher positionality, and potential misinterpretation of findings, a research team member [PK (PhD, male, extensive research experience with a strong focus on experimental Natural Sciences, with some experience on qualitative methodologies of Social Sciences)] separate from the facilitators reviewed findings to ensure credibility (Male, 2016). The research team members had previously met and collaborated with some of the virtual marketplace participants prior to the research study.

## Results

This section groups the results based on the study procedure of hammer and anvil, hurdle and magnifying glass from the rapporteur whiteboard notes and as summarized in Figure 1 (Haven and Lacey, 2024).

### Hammer and anvil: what works well for successful implementation?

Participants echoed three main practices for ensuring training works well. First, training must be tailored. This meant it would be designed around real cases and use active learning approaches that promote reflection (e.g., role-play or forms of peer-to-peer intervision) and are taught in groups as opposed to individuals. There was agreement among participants that “…*the real issue is engaging [supervisors and leaders] in the discussion. Quality of the course. Discussion of real cases. Relevance for their teaching/supervision*”. Second, training would be embedded within the (institutional) implementation of RI policies, meaning it would align with training directed at other levels of seniority and link to existing issues that institutions also seek to provide training for, including data management and open science. Relatedly and thirdly, training implementation must be aligned with institutional expectations around what it means to be a good researcher and that “*senior and early researchers [need to] understand that being a good researcher doesn't make you a good supervisor*”.

### Hurdle: what are the challenges?

Key challenges discussed included the difficulty with getting senior supervisors to take part in training programs. Even if mandated, having seniors attend but not engage does not promote responsible research. Relatedly, some participants recounted training initiatives that were met with resistance, because of a perceived lack of time, could be countered with “*shared [supervision], [and] limits on number of students per supervisor*”. Participants also mentioned the widespread belief that intending to do responsible research is sufficient and that “*individuals need to understand that wanting to do good research is not enough and that unwilling errors/biases are an issue (as Daniel Barr said: difference between trustworthiness of individuals and of research)*”. Whereas they knew of many instances where this could result in outdated knowledge about issues in RI, in particular in quickly evolving issues such as artificial intelligence with a “*lack of clear guidelines on what is good practice*”. Some participants indicated that current training initiatives are scattered, oftentimes only a one-off in the form of a seminar, occurring once in the duration of a semester, which makes it difficult to determine their effectiveness and promote scaling up. This ties into the broader challenge of valuing and recognizing good research practices and good supervision on an institutional level as “*good research [is] not part of [the] criteria for researchers*—*not recognised by institutions*—*funding bodies don't recognise good researchers, good supervisors*”. Finally, without consistent support from senior management, it was difficult to get training initiatives off the ground.

### Magnifying glass: how do we assess effectiveness?

In terms of meaningful ways to assess the effectiveness of implementing good practice for training supervisors and leadership, participants stated that first before assessing, supervisors and leaders should be clear as to what good practice is and who is deciding, as “*good supervision practice is subjective*”. Some participants felt that how to assess could be from learning from external stakeholders, then sharing that knowledge internally, by building a community of practice (CoP) amongst the supervisors and leadership, to create a platform and environment for shared learning. Other methods for assessing effectiveness were around engaging with the open science practices of pre-registration, sharing research data and open access. This would have the added benefit for “*countries with no structures for promoting RI in general*”. Some participants were strong advocates for peer review to learn from others and being a mechanism to assess own effectiveness. Participants also shared opinions on leveraging surveys to assess effectiveness longitudinally, while being cognizant of the need for 360° of feedback for respondents that engage with the survey. Finally, it was mentioned that institutions should treat incidents of research misconduct as “*an opportunity for learning*”, where there is transparency around the implementation of the procedure, with the appropriate levels of anonymity, and openness to the outcomes of such incidents, to enable learning from researchers and awareness that incidents do happen and are managed fairly, consistently and thoroughly, with the potential of developing a “*trust-based open environment rather than a policy[ing environment]*”.

## Discussion

A virtual marketplace was facilitated as a platform for exchange of current practices and approaches for training supervisors and leadership to gather evidence on (1) what works well for successful implementation, (2) what are the challenges, and (3) how we assess effectiveness. Participant inputs and experiences to the three questions were interrelated with agreement on the need for training to be tailored, practical oriented, and mandated, while cognizant of hurdles around: (a) being clear as to what good research practice is and who is deciding, (b) that senior researchers resist taking part due to a lack of time to engage. Additionally, participants were clear that for sustainability and impact, training needs to be supported by senior management, become embedded in the institutional research culture, and move beyond an intention to doing responsible research. With the ever-evolving research landscape, participants stated there is a constant need to learn, from stakeholders, incidents of research misconduct and feedback surveys, along with engagement in peer review and open science practices.

### Contextualization

In the literature the pressure to publish remains a staple to success in research (Neill, 2008; Rawat and Meena, 2014). Data gathered from the virtual marketplace stresses the need for training supervisors and leadership to value quality over quantity, limiting the number of students per supervisor, and engaging with good research practices. Considering the limited literature for research supervisors' and leaders' training and evidence around what works, this current study provides inputs from the perspectives and experiences of European research policy makers, supervisors and senior research leaders, in terms of: (a) mandating training, (b) setting training in small groups and for different levels, (c) embedding training in institutional policies and research strategies. With a potential move to valuing quality over quantity, maybe there is an opportunity to leverage alternative metrics including but not limited to patents, contributions to policy documents, as they can be used for impact measurement, public engagement and adherence to open science and transparency practices (Szomszor and Adie, 2022; Franzen et al., 2023).

The SOPs4RI project (Tijdink et al., 2023) has developed guidelines on building and leading an effective team^3^ and on responsible supervision^4^ that target the institutional level of RPOs. In the former set of recommendations there is a clear mention that responsible leadership can be promoted by training research leaders on RI, while in the latter set of recommendations there is the statement on the positive spillover effect of policies and guidance on supervision on raising awareness about RI and responsible supervision. In addition, the guidelines for research institutions on continuous RI education,^5^ developed by SOPs4RI, bring forward that “*training is an important aspect of research integrity education*”, with the note that RI education with a continuous character requires informal approaches, such as—among others—learning by doing. Such guidelines are clearly in line with the results of this study; they describe the need of RI education and training for supervisors and research leaders, while providing the need for a similar problem-based learning approach.

An extension to one of the Wellcome Trust (2020) findings around leadership needing to be transparent while also being a mechanism for assessing effectiveness is around engaging with the open science practices of pre-registration, open data and open access. A positive implication to engaging with open science practices would be alignment with reforming research assessment initiatives of DORA (Declaration on Research Assessment, 2012), the Leiden manifesto (Hicks et al., 2015), the Hong Kong Principles (Moher et al., 2020), and CoARA (Science Europe, European University Association, and Stroobants, 2022). However, a current hurdle when engaging with open science practices and learning from external agencies is that this is often done on supervisors and leadership free time, placed upon an already intense workload, and requiring funding support to consider the cost of article processing charges. However, there is low hanging fruit in terms of minimal cost to pre-registering studies and sharing data openly.

Hanover Research (2014) and Senge et al. (2014) speak to the importance of collective leadership and consistency when trying to instill change in HEIs, however, as it currently stands, without formal recognition through research assessment approaches, training will be *ad hoc* and inconsistent across HEIs. Furthermore, training is simply not sustainable if being done in supervisors and leadership's free time and relatedly, if promotion of RI is being driven by one individual. Again, this speaks to the need to have training embedded in the research culture. Of course, a hurdle with embedding RI in a HEI's/RPO's research culture is that a culture often is HEI/RPO and country specific. Some HEIs/RPOs/countries may be quite proactive while others may not have the appropriate funding and support and thus may fall behind. A potential interim bridge to address this inequity is again around recognizing and rewarding open science practices.

## Limitations and future research

A key limitation to this study is the small sample size and with 22 participants across 13 European countries there is the potential for biases through self-reporting which may not be replicable nor generalizable. Another limitation is the absence of participants from some European countries, although there were specific recruitment efforts, full European coverage was not obtained, with the potential of a Western European bias in the findings. Also, the study did not include PhD candidates and junior researchers which is certainly worth considering for future research, and depending on the scope of a future study, the inclusion of undergraduate students would be warranted. These perspectives are crucial when discussing hierarchical dynamics and power imbalances in good research practices. To maintain participant anonymity minimal demographic information could be utilized. If the study were larger, additional demographic information would be analyzed to allow greater synergy between qualitative and quantitative analysis. For a broader study on training supervisors and leadership, a mixed-methods approach could be advantageous, while an increase in sample size could seek to include several practitioners in each region to allow for comparison within and across regions, along with the inclusion of underrepresented regions However, with minimal evidence in the literature on the current study's topic, there was no secondary data available that could be used to explore hypotheses. This, combined with the exploratory goals of the study, meant the selected methods were deemed appropriate for initial insights to inform future research (Connolly, 2016).

Another point to explore in future research is how problem-based learning may truly foster change in supervisor behavior. While this study presents findings on the need for training being tailored to specific disciplines and conducted in small groups using a problem-based learning approach, it would be worth following up on this need with evidence that the approach is effective in leading change. One concrete pathway to operationalize CoARA's emphasis on the importance of acknowledging a wide range of scholarly contributions, including mentoring, supervision, leadership, and training, as integral to academic performance, would be to include the successful completion of supervisor training in internal promotion criteria. On a broader, institutional level, the percentage of senior staff that has completed supervision training could be mapped on an institutional dashboard (similar to what Franzen et al., 2023 did when creating institutional dashboards for data sharing and trial registration), directly underscoring the institution's commitment to CoARA's principles, and its active monitoring thereof. If applied on a country-wide level, this would allow PhD applicants to choose their institution and supervisor based on its commitment to ensuring a responsible and open research environment.

Leadership metrics in diverse work environments highlight the need for greater emphasis on cultural respect, societal values, and practical strategies for driving positive change (Macfarlane et al., 2024). While attributes such as knowledge sharing (Sonmez Cakir and Adiguzel, 2020) and interpersonal accuracy (Dael et al., 2022) align with participant insights from this study, they are distinct and warrant further exploration in future research.

## Conclusion and a roadmap to good practice for training supervisors and leadership

With the findings from this research study based on inputs from 22 research policy makers, supervisors and senior research leaders across 13 countries, there is an opportunity to posit a roadmap to good practice for training supervisors and leadership. The marketplace exchange indicates that stakeholders across Europe agree on the urgency with which RI training should be mandated, tailored to specific disciplines and conducted in small groups, using a problem-based learning approach. The next step involves structured pilots that are rigorously evaluated. As part of this evaluation, PhD candidates and others who receive leadership must be involved. The status quo is that too often only leaders and supervisors, who are already motivated, participate in training. Some supervisors and research leaders are reluctant to participate in RI training, based on the arguments that current training programmes are unscientific and due to lack of time. To ensure sustainability of the training and being part of the research culture, the training requirement should be clearly stated in institutional policies with regular review of the policies, given the ever-changing research landscape and tailored RI-related training interventions that are developed. To overcome resistance from senior supervisors and leaders to participate and support research training, the training should be formally recognized, not only as mandated by institutional policy, but also as a formal component of continuous professional development and research assessment. There is an opportunity that the training could overlap with the promotion and encouragement of open science practices. Finally, when engaging with end-users and soliciting feedback, closing the feedback loop and informing end-users of the impact of their feedback is very important.

## Data Availability

The datasets presented in this article are not readily available because to support the research outputs from the marketplace, a minimal amount of demographic information on participants was obtained. As part of the informed consent we made participants aware that the responses they provide will be potentially identifiable to the authors, but when the information is disseminated more widely, all efforts will be made to ensure anonymity. Requests to access the datasets should be directed to sean.lacey@mtu.ie.

## Footnotes

^1^ https://nerq.eu/

^2^ https://www.gather.town/

^3^ https://osf.io/qbpc9

^4^ https://osf.io/4w89m

^5^ https://osf.io/ambg3

## Appendix

A minimal amount of demographic information on participants for context, was solicited through an anonymous survey before the marketplace.

## Introduction

Dear colleague,

Thank you for agreeing to participate in the *Virtual Marketplace on Good Practice for Training Supervisors and Leadership*. As mentioned in the invitation letter, we aim to disseminate the outputs from the marketplace. To support the outputs from the marketplace it would be beneficial to provide a minimal amount of demographic information on marketplace participants. We would like to gather this information in the following anonymous form.

You may withdraw while completing the form by simply closing the internet window that the form is on. We are aware that the responses you provide will be potentially identifiable to us, but when the information is disseminated more widely, all efforts will be made to ensure your anonymity.

Data will be stored securely in accordance with the General Data Protection Regulations and the aggregated summary of results may later be used in social media posts, along with being published for research/educational purposes. Data will be stored securely in password protected files for 1 year after final data collection date. After this, anonymized data will be destroyed.

With the exception of the informed consent question in the form, responses to questions are optional. The form should take <5 min to complete.

This study has received ethical approval from Munster Technological University (Human Research Ethics Approval No: MTU-HREC-MR-23-058-A).

If you have any further questions about the form and/or the research study, you may contact Dr. Seán Lacey (sean.lacey@mtu.ie). If you wish to make a complaint, please send an email with details to the Human Research Ethics Committee, Munster Technological University, on hrec@mtu.ie so that they can look into the issue and respond to you.

Kind regards,

Tamarinde Haven, Seán Lacey.

## Questions

1. Please confirm below that you have read the information above and consent to the data being used in the way described.
  ° Yes, I consent to my responses being used as described above. (Survey moves to Q2.)
  ° No, I do not consent to my responses being used as described above. (Survey closes.)
2. Please state your role title. (Open-ended question.)
3. In your current role, do you have time to carry out research?
  ° Yes
  ° No
4. What European country is your main place of work? (Open-ended question.)
